# Targeted Control of Chloroplast Quality to Improve Plant Acclimation: From Protein Import to Degradation

**DOI:** 10.3389/fpls.2019.00958

**Published:** 2019-07-25

**Authors:** Xiaolong Yang, Yangyang Li, Mingfang Qi, Yufeng Liu, Tianlai Li

**Affiliations:** Key Laboratory of Protected Horticulture of Ministry of Education, National & Local Joint Engineering Research Center of Northern Horticultural Facilities Design & Application Technology (Liaoning), Horticulture Department, Shenyang Agricultural University, Shenyang, China

**Keywords:** chloroplast protein import, selective autophagy, ubiquitin-26s proteasome system, chloroplast retrograde signaling, environmental stress, chloroplast homeostasis

## Abstract

The chloroplast is an important energy-producing organelle acting as an environmental sensor for the plant cell. The normal turnover of the entire damaged chloroplast and its specific components is required for efficient photosynthesis and other metabolic reactions under stress conditions. Nuclear-encoded proteins must be imported into the chloroplast through different membrane transport complexes, and the orderly protein import plays an important role in plant adaptive regulation. Under adverse environmental conditions, the damaged chloroplast or its specific components need to be degraded efficiently to ensure normal cell function. In this review, we discuss the molecular mechanism of protein import and degradation in the chloroplast. Specifically, quality control of chloroplast from protein import to degradation and associated regulatory pathways are discussed to better understand how plants adapt to environmental stress by fine-tuning chloroplast homeostasis, which will benefit breeding approaches to improve crop yield.

## Introduction

As sessile organisms, the ability to quickly sense and adapt to their surroundings is essential for plant survival. Understanding stress signaling and response will enable us to improve stress resistance in crops, which are critical to agricultural productivity and sustainability in changing environments. The stress-generated signals in organelles can regulate stress-responsive gene expression and cellular activities to maintain cellular homeostasis (Zhu, 2016). Many previous studies have focused on photoinhibition and photoprotection mechanisms of plants growing in fluctuating environments; however, the turnover of chloroplast proteins is also essential for maintaining efficient photosynthesis and metabolism. A comprehensive understanding of how chloroplast maintains homeostasis will provide detailed insights for plant resistance and crop improvement. In this mini-review, we first introduce the most important recent publications which revealed the molecular mechanism of chloroplast protein import and then review chloroplast protein degradation processes including proteolysis, the ubiquitin-proteasome system (UPS), the newly confirmed autophagy process, and other related pathways (Ling and Jarvis, 2016; Izumi et al., 2017; Chen et al., 2018; Ganesan et al., 2018; Kikuchi et al., 2018). Finally, we discuss biological processes related to chloroplast quality control, in order to enhance plants’ adaptability to the fluctuating environments.

## The Maintenance of Chloroplast Homeostasis Involves Protein Import and Degradation

The chloroplast is a complex organelle that contains three membrane systems – the outer envelope, the inner envelope, and the thylakoid membrane; as well as three major sub-compartments – the intermembrane space between the outer and inner envelope, the stroma, and the thylakoid lumen (Nakai, 2018). Chloroplast division is accomplished by the constriction of plastid division machinery, which is essential for maintaining an optimal plastid number in plant cells (Gao et al., 2013; Wang et al., 2017a,b). The chloroplast genome encodes less than 100 proteins and most proteins (about 95%) in the chloroplast are encoded by nuclear genes. Nuclear gene-encoded proteins must be translocated to the chloroplast for its biogenesis and functional maintenance.

Abiotic stresses such as strong light, CO_2_ limitation, or extreme temperatures can induce the photoinhibition of PSII and PSI and impose continuous oxidative damage to the chloroplast during energy production, which further inhibit photosynthesis efficiency and plant growth (Dietz et al., 2016; Malnoë, 2018). Proteins in the photosynthetic electron transport chain are vulnerable targets of oxidative damage. All photodamage and photoprotection responses involve the rapid turnover of specific photosynthetic proteins (Li et al., 2018). Recent protein turnover studies found that PetD (a subunit of the cytochrome b6f complex), PIFI (an auxiliary subunit associated with the NDH complex), NAD(P)H dehydrogenase subunit K (NDHK), zeaxanthin epoxidase (ZEP), and proton gradient regulation-like 1 (PGRL1) have rapid import and degradation rates (Li et al., 2017). Together with the discovery of photodamage-induced chlorophagy, these results indicate that the photosynthetic electron transport chain is essential for chloroplast quality.

Many biological processes need to be strict yet flexibly controlled in the chloroplast because the functional proteins are encoded by two separated organelles – the nucleus and the chloroplast. Light signals, developmental stage, hormones, metabolites, and environmental changes all can lead to transcriptional reprogramming in the chloroplast (Wang et al., 2017a,b). Communication between chloroplast and the nucleus under stress conditions is closely connected to chloroplast protein import and degradation. Many biological processes in chloroplast are regulated by nuclear factors (anterograde signals), while the transcription of some nuclear genes is sensitive to signals from the developing chloroplast (retrograde signals).

## The Entrance of Chloroplast Protein Import is Precisely Controlled

Proteins are imported into the chloroplast through four major steps: (1) specific sorting of a precursor protein in the cytoplasm; (2) recognition by the receptor at the outer membrane of the chloroplast; (3) consecutive translocation across both membranes; and (4) accurate processing and sorting after the protein is imported (Lee et al., 2017). Precursor proteins interact with many cytoplasmic molecular chaperones to form guidance complexes; transmembrane transport is launched once these complexes are recruited by the import receptor at the chloroplast envelope (Nakai, 2018). Precursor proteins usually contain a chloroplast transit peptide (cTP) – a cleavable N terminal signaling sequence that guides the preproteins target to chloroplast. cTPs can be removed by a stromal processing peptidase (SPP), while the thylakoid-transfer signal (TTs) is cleaved by thylakoid processing peptidase (TPP). Each transit peptide usually contains motifs that can interact with different translocon components; thus, cTP determines plastid types, precursor protein selectivity, and import pathways in different developmental stages and environmental conditions (Li and Teng, 2013). A site-specific crosslinking approach to map the topological structure of transit peptides revealed that the signal peptides were sequentially recognized by the translocon on the outer (Toc) and inner (Tic) envelope supercomplexes during protein import (Richardson et al., 2018).

**Figure 1:**
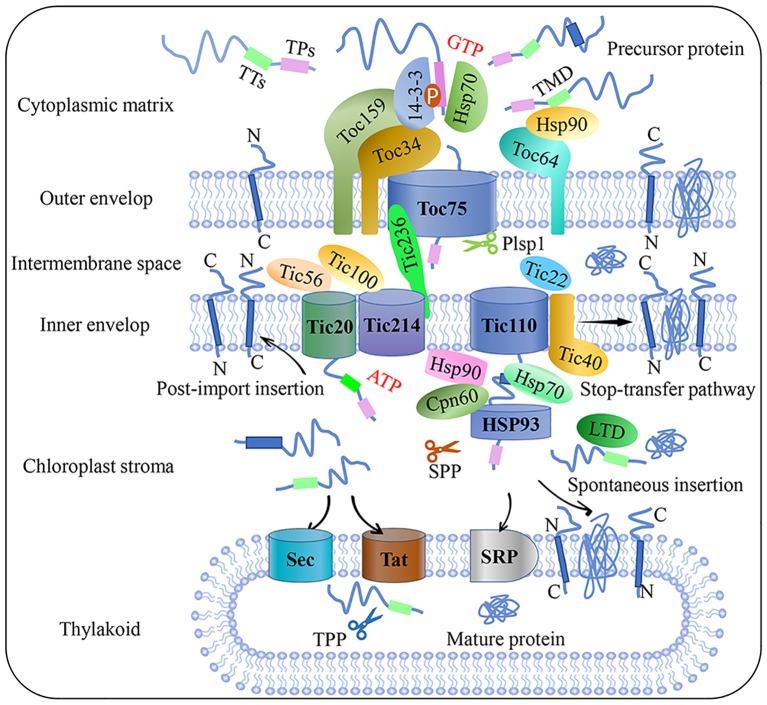
Flow chart of plant chloroplast protein import. Precursor protein entered the intermembrane space through the Toc complex after recognition by the guidance complex. With the aid of molecular chaperones and stroma processing peptidase, precursor protein is then translocated into the chloroplast stroma through the Tic complex. Preproteins target the inner membrane through post-import insertion and stop-transfer pathways. Preproteins can also be inserted into the thylakoid membrane through SRP or spontaneous insertion and translocation across the thylakoid membrane through the Sec and Tat complex, respectively. Plsp1: plastid type I signal peptidase 1; Sec: secretion; SPP: stromal processing peptidase; SRP: signal recognition particle; Tat: twin-arginine translocation; Tic: translocon at the inner envelope membrane of chloroplasts; TMD: transmembrane domain; Toc: translocon at the outer envelope membrane of chloroplasts; TPs: transit peptide sequence; TTs: thylakoid targeting sequence.

The Toc-Tic supercomplex is generally considered to be the gateway for most chloroplast proteins (Figure 1; Chen and Li, 2017). The Toc complex dominates the initial recognition and translocation of preproteins at the outer membrane. Toc core proteins include the membrane channel protein Toc75, and two GTPase receptors, Toc34 and Toc159. The cTP of a precursor protein can be specifically recognized by Toc34 and Toc159 before the precursor protein is transported by the channel protein Toc75. The polypeptide transport-associated (POTRA) domains of Toc75 can directly interact with cTP to facilitate import (Chang et al., 2017; O’Neil et al., 2017). Moreover, Toc64 functions as an essential transport component with dual function. Toc64 can recognize Hsp90 and deliver precursor proteins *via* a cytosolic exposed domain that contains three tetratrico-peptide repeat motifs, and also functions as a major component of a complex facing the intermembrane space (Qbadou et al., 2007; Tillmann et al., 2015). As a molecular chaperone in the chloroplast intermembrane space, Tic22 can interact with the precursor protein to ensure correct targeting during its transport from the Toc complex to the Tic complex (Rudolf et al., 2013). Research on components of the Tic complex is controversial; the classical model of the Tic complex generally assumes that Tic40 can directly interact with Tic110 and the stroma molecule chaperone to assist transmembrane transport (Flores-Pérez and Jarvis, 2013; Bédard et al., 2017). However, this model was called into question by Nakai and associates, who proposed the Tic20/Tic56/Tic100/Tic214 complex as the main import route of chloroplast inner membrane transport (Kikuchi et al., 2013). Recently, a 2-MD heteromeric AAA-ATPase complex was identified as the import motor pulling preproteins across the inner envelope (Kikuchi et al., 2018). Chen et al. (2018) identified a new Tic component, Tic236, which is anchored to the inner membrane and binds directly to the outer-membrane channel Toc75. This long and stable protein bridge between Tic and Toc complex is necessary for protein translocation into the chloroplast. A recent study found that the functional pore size of the Toc-Tic complexes is greater than 25.6 Å and the pores have a strong unfoldase activity, providing flexibility and expandability to accommodate and import folded proteins into the chloroplast. This unique character is essential for controlling chloroplast protein import (Ganesan et al., 2018).

The chloroplast protein import apparatus must be dynamically and precisely regulated to control translocation efficiency and the abundance of new protein sets. Reversible phosphorylation is emerging as an important regulatory mechanism during cytosolic sorting and targeting of chloroplast preproteins prior to translocation. Recent studies reported that kinase of the outer chloroplast membrane 1 (KOC1) can phosphorylate the A-domain of the import receptor Toc159 *in vitro* and accelerate precursor protein import (Zufferey et al., 2017). Furthermore, a relationship between the degradation of chloroplast outer envelope proteins and the UPS has come to light in recent years. This proteolytic system is referred to as chloroplast-associated protein degradation, or CHLORAD, which involves SP1 (suppressor of ppi1 locus 1 E3 ligase), SP2 (an Omp85-type b-barrel channel of the OEM), and CDC48 – a cytosolic AAA+ (ATPase associated with diverse cellular activities) chaperone, is vital for organelle functions and plant development (Ling and Jarvis, 2015; Ling et al., 2019). In addition, photosynthetic electron transport in the chloroplast is a dynamic process of oxidation and reduction, so the fluctuating redox state can affect either the activity of the Tic channel or the binding capacity of both the Toc and Tic translocation apparatuses at different stages of import, especially under adverse conditions. Though Toc-Tic supercomplexes is the general import pathway, the recognition can be lack specificity as seen in dual-targeted proteins transport process, in addition, other alternative protein import pathways were found, such as the transit peptide-independent import pathway and protein deliver from the ER/Golgi apparatus to the chloroplasts. More detailed overviews on chloroplast protein import and related regulatory pathways can be found in several recent reviews (Li and Chiu, 2010; Lee et al., 2017).

## Internal Degradation of Damaged Chloroplast Components *via* Proteolysis

More than 20 proteolytic enzymes have been found to hydrolyze chloroplast proteins, including the caseinolytic protease (Clp), degradation of periplasmic (Deg) protease, filamentation temperature-sensitive H (FtsH), organellar oligopeptidase (OOP), presequence peptidases (PreP), and chloroplast nucleoid DNA-binding protein 41 (Cnd41) (Nishimura et al., 2017). The proteolytic machinery is widely involved in the import of newly synthesized chloroplast proteins and degradation of damaged components. Upon import into the chloroplast, cTPs of the preproteins are cleaved off by stromal processing peptidase (SPP) and the cleaved cTP fragments are then degraded by PreP and OOP (Nishimura et al., 2017). The proteolytic subunit 3 of Clp protease could degrade damaged chloroplast proteins and recycle amino acids in dark-treated *Camellia sinensis* leaves (Chen et al., 2017). The downregulation of genes encoding Clp subunits can affect chloroplast development and cause abnormal plant or embryo development (Kim et al., 2015; Nishimura and van Wijk, 2015). Deg and FtsH proteases are crucial for the photodamage repair cycle by accelerating the turnover of photosystem II reaction center protein D1 and cytochrome *b6f* complex, which plays a key role in regulating photosynthesis and photoprotection (Malnoë et al., 2014; Kato et al., 2018; Knopf and Adam, 2018).

## Multiple Extraplastidic Degradation Processes of the Whole Chloroplast and its Specific Components

Ubiquitin is an essential signaling molecule for the selective degradation of proteins, which depends on the UPS (Hua and Vierstra, 2016). UPS can specifically identify and degrade protein substrates through E3 ligase. Proteomic analysis revealed that the 26S proteasome subunits RPT2a/b and RPT5a interact directly with the transit peptides of three chloroplast proteins in the cytosol, and are essential for the degradation of unimported chloroplast protein precursors (Sako et al., 2014). Under certain conditions, the *Arabidopsis ferrochelatase 2* (*fc2*) mutant accumulates excessive amounts of protoporphyrin IX and ^1^O_2_ in the chloroplast. Thereby, damaged chloroplasts are tagged with ubiquitin for selective degradation, which is accelerated by plant U-box (PUB4) E3 ligase (Woodson et al., 2015). The SP1 E3 ligase mediates the degradation of specific components of the Toc complex *via* 26s proteasomes under moderate stress conditions, while under severe stress, the PUB4 E3 ligase mediates the ubiquitination of chloroplast membrane proteins and subsequent degradation of the entire damaged chloroplast (Ling and Jarvis, 2016).

**Figure 2:**
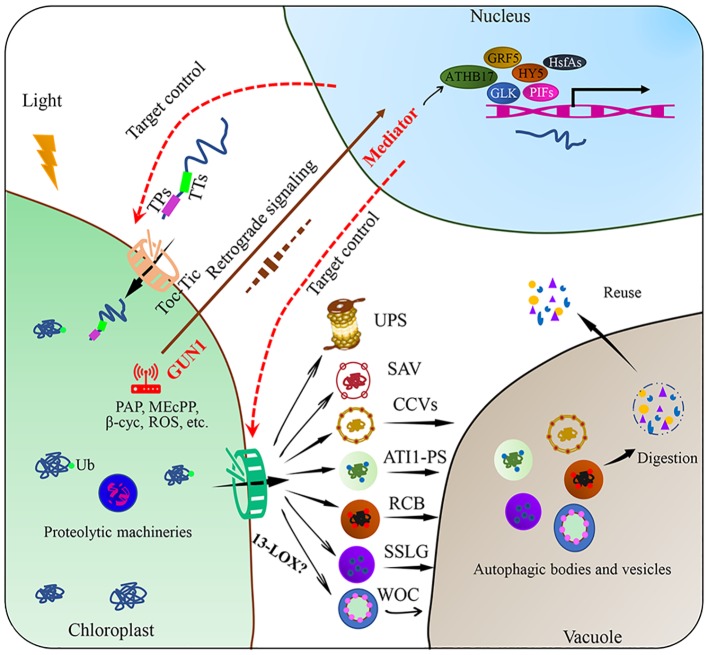
Targeted control of plant chloroplast quality. Chloroplast proteins encoded in the nucleus are transported across the chloroplast envelope through Toc-Tic complexes. Chloroplast protein degradation through various pathways, including chloroplast protein hydrolysis and the SAVs, CCVs, ATI1-PS, RCB, SSLG, or WOC, these degraded substances can be reused within the cell after degradation. Chloroplast retrograde signaling is involved in the regulation of chloroplast proteome remodeling under fluctuating environmental conditions. Plastid metabolism includes phosphonucleotide 3′-phosphoadenosine 5′-phosphate (PAP), methyl erythritol (MEcPP), beta cyclocitral (beta cyc), fatty acids (FAs), and ROS. After environmental stimuli are perceived by the chloroplast, some transcription factors, such as PIFs, GLK, HY5, GRF5, HsfAs, and ATHB17, are involved. Chloroplast protein import and export are the potential targets of chloroplast quality control. Ub: ubiquitin; RCBs: RuBisCO-containing bodies; ATI1-PS: ATG8-interacting protein 1 positive bodies; SSLGs: small starch-like granule bodies; SAVs: senescence-associated vacuoles; CCVs: chloroplast vesiculation-containing vesicles; PIFs: phytochrome interaction factors; HY5: elongated hypocotyl 5; GLK: golden 2-like, ATHB17: *Arabidopsis thaliana* homeobox17, GRF5: growth retardation factor 5; HsfAs: heat stress transcription factors.

Autophagy is a ubiquitous macromolecular degradation process in eukaryotic cells mediated by proteins encoded by autophagy-related genes (ATGs) (Marshall and Vierstra, 2018). RuBisCO-containing bodies (RCBs), ATG8-interacting protein 1 positive bodies (ATI1-PS), and small starch-like granule bodies (SSLGs) are responsible for the selective autophagy of chloroplast components and the entire damaged chloroplasts (Figure 2; Otegui, 2017; Izumi and Nakamura, 2018). The formation and transport of RCBs depend on the cellular carbon status and can be inhibited by light or sugar addition in the substrate, indicating that RCBs can recycle energy and carbon from stroma proteins and especially the small subunits of RuBisCO in energy-constrained conditions (Ishida et al., 2014). Carbon starvation-induced ATI1 expression can cause selective autophagy of specific plastid proteins, which are eventually transferred to the vacuole for degradation (Michaeli et al., 2014). In addition, Izumi et al. (2017) reported that photooxidative damage-induced chlorophagy can transport the entire chloroplast to the vacuole for degradation. Further studies revealed that the induction of chlorophagy can be mediated by chloroplast swelling due to high light-induced envelope damage and executed *via* tonoplast-mediated sequestering (Nakamura et al., 2018). This entire-organelle-type chloroplast autophagy is different from piecemeal chlorophagy, which involves the removal of small membrane vesicles from the organelle (Otegui, 2017).

Other extraplastidic degradation pathways through senescence-associated vacuoles (SAVs) and chloroplast vesiculation-containing vesicles (CCVs) have also been discovered (Otegui et al., 2005; Wang and Blumwald, 2014). SAVs have higher proteolytic activity and acidity than the central vacuole. SAVs accumulate in senescing leaves and are involved in the degradation of soluble photosynthetic proteins in the chloroplast stroma (Otegui et al., 2005; Martínez et al., 2008). Unlike autophagy, SAVs can degrade chloroplast components, while how SAVs are formed and how the chloroplast components are translocated to SAVs remain unknown (Carrión et al., 2013). A recent study has illustrated that SAVs have strong cysteine protease activity and the most widely used senescence-associated protein SAG12 may participate in RuBisCO breakdown during leaf senescence (James et al., 2018). Chloroplast degradation through CCVs is independent of autophagy and SAVs. After proteins are transported to the chloroplast, chloroplast vesiculation (CV) destabilizes the chloroplast and induces the formation of CCVs during senescence or under abiotic stress conditions, further delivering chloroplast proteins to the vacuole for proteolysis (Wang and Blumwald, 2014). Recently, researchers found that CV-related gene expression was induced by the NAC (NAM, ATAF1/2, and CUC2) transcription factor RD26, which directly regulates the degradation of chloroplast proteins during senescence (Kamranfar et al., 2018). Under water-limited conditions, the expression of *OsCV* increased in wild-type plants, whereas *OsCV*-silenced plants exhibited an improved carbon and nitrogen fixation efficiency and environmental acclimation (Sade et al., 2017).

## Retrograde Signaling and Transcriptional Control of Chloroplast Composition

Chloroplast protein import especially through the Toc-Tic supercomplexes and multiple degradation machineries function as key control targets of chloroplast quality. Chloroplast protein import is highly dependent on developmental stages and environmental factors. The operating efficiency of the import channel is regulated by redox state, phosphorylation, ubiquitylation, and molecular chaperones in the cytoplasm and stroma (Woodson, 2016). Autophagy and vesicles are the final steps in the degradation of chloroplast and its constituents, while the initial steps remain unclear. A previous study showed that 13-lipoxygenase (13-LOX), which targets the chloroplast, induces programmed chloroplast injury. Once imported into the chloroplast, 13-LOX generates holes in the chloroplast envelope by oxidizing unsaturated fatty acids in the chloroplast membrane, allowing the delivery of stroma proteins across the chloroplast envelope (Springer et al., 2016).

Chloroplast retrograde signaling, which triggers a precise spatial transcriptional regulation trade-off between protein import and degradation, could be an important strategy to control chloroplast quality. As the main communication language between the chloroplast and nucleus, operational chloroplast retrograde signals can regulate energy allocation between stress acclimation and growth by coordinating transcriptional reprogramming to control chloroplast quality (Watson et al., 2018). The most studied chloroplast retrograde signals include methyl erythritol (MEcPP), phosphonucleotide 3′-phosphoadenosine 5′-phosphate (PAP), beta cyclocitral (beta cyc), fatty acids (FAs), and ROS (Chi et al., 2015; Benn et al., 2016; Dietz et al., 2016; Page et al., 2017). These stress-triggered chloroplast retrograde signals must be integrated with the cytosolic stress signaling network, transduced to the nucleus and connected to transcriptional regulators. For example, genomes uncoupled 1 (GUN1) may act as a central hub in the chloroplast by integrating different retrograde signals; whereas the mediator of transcriptional co-activator complex may function as a regulatory hub in the nucleus by integrating complex stress signaling networks to control the transcriptional remodeling required for stress acclimation (Crawford et al., 2017; Hernández-Verdeja and Strand, 2018).

Epigenetic modification and transcriptional regulatory factors may be activated to maintain the stability of the chloroplast proteome after sensing special environmental stimuli. Redox metabolism through key intermediates in the chloroplast is closely linked to numerous epigenetic markers and stress responses (Locato et al., 2018). DNA methylation in the promoter region of *OsAK1* caused albinism in young leaves and abnormal chloroplast development in rice (Wei et al., 2017). DNA methylation and histone acetylation that regulate photosynthesis and development are conserved in *Arabidopsis thaliana* and *Populus trichocarpa* (Valledor et al., 2015). Chloroplast signals participate in epigenetic controlling the MUTS HOMOLOG1 (MSH1) protein targeted to both the mitochondria and chloroplasts, fulfilling essential functions in control of plant development (Virdi et al., 2016). Transcription factors specifically regulate chloroplast development, and expression of the photosynthesis-related genes is induced by photoreceptor-mediated light signal related transcription factors, such as the phytochrome interaction factors (PIFs) and elongated hypocotyl 5 (HY5) (Kharshiing and Sinha, 2016; Wang et al., 2017a,b). In addition, golden 2-like (GLK), *Arabidopsis thaliana* homeobox 17 (ATHB17), growth regulation factor 5 (GRF5), and heat stress transcription factors (HsfAs) can integrate different developmental, hormonal, or environmental signals to control photosynthetic gene expression and chloroplast development (Vercruyssen et al., 2015; Leister and Kleine, 2016; Zhao et al., 2017). Further studies are required to better understand the molecular mechanism of chloroplast signals and transcription factors in the regulation of chloroplast protein import or degradation. How photosynthesis and metabolism control chloroplast quality under abiotic stress should also be investigated.

## Conclusions and Perspectives

Environmental stress induced oxidative damage to the chloroplast and its components significantly decrease photosynthetic efficiency and crop yield. The rapid turnover of the entire chloroplast and its specific components is crucial for maintaining photosynthesis and metabolism but the relationship between photoinhibition and chloroplast quality under stress conditions require further clarification (Li et al., 2017; Tamburino et al., 2017). We propose that the targeted control of chloroplast quality through regulating the channels by which proteins are imported and exported across the membranes can be used to improve plant acclimation to changing environments (Figure 2). The advanced and widely used genome editing technology can be utilized to control chloroplast quality through precise genetic modification of import and export channels and related transcriptional regulatory pathways, and we believe it is an important future research direction especially in the field of plant stress biology. However, the potential effects of targeted control of chloroplast homeostasis on the adaptability to fluctuating environments of plants and improved production in major crops require further evaluation.

## Author Contributions

XY, YLiu, and TL are responsible for the general overview of the opinions stated in the manuscript. XY and YLi wrote and modified the manuscript. MQ reviewed and modified the manuscript. All authors reviewed and approved the final version of the submitted manuscript.

### Conflict of Interest Statement

The authors declare that the research was conducted in the absence of any commercial or financial relationships that could be construed as a potential conflict of interest.
